# DO NEARBY PHYSICAL ACTIVITY DESTINATIONS ENCOURAGE PEOPLE TO BE F.I.T.T.?

**DOI:** 10.1093/geroni/igad104.0018

**Published:** 2023-12-21

**Authors:** Patrick Donahue, Kyle Moored, Breanna Crane, Emily Richards, Michael Desjardins, Andrea Rosso, Michelle Carlson

**Affiliations:** Johns Hopkins University, Baltimore, Maryland, United States; Johns Hopkins Bloomberg School of Public Health, Baltimore, Maryland, United States; Johns Hopkins Bloomberg School of Public Health, Baltimore, Maryland, United States; Johns Hopkins University, Baltimore, Maryland, United States; Johns Hopkins Bloomberg School of Public Health, Baltimore, Maryland, United States; University of Pittsburgh, Pittsburgh, Pennsylvania, United States; Johns Hopkins Bloomberg School of Public Health, Baltimore, Maryland, United States

## Abstract

Neighborhood amenities may reduce barriers to engaging in physical activity (PA). However, it is unclear whether proximity to these amenities will indeed promote increased PA. This study examined whether living near PA destinations was associated with greater self-reported PA. The sample included Cardiovascular Health Study participants (N=3,922; mean age=75.3 years) from visit 5 (1992-1993). Quantity of PA destinations (e.g., gyms) within a one-kilometer Euclidean buffer of each participant’s home address was provided by the National Establishment Time-Series database. PA over the previous two weeks was self-reported via the Minnesota Leisure Time Activity Questionnaire. The exposure was dichotomized into having at least one PA destination (vs. none) within one kilometer of participants’ homes. Outcomes were based on the FITT components of PA outlined by the American College of Sports Medicine: Frequency (activities per week), Intensity (MET-minutes per week), Time (minutes per day), and Type (number of different activities, i.e., variety). Outcomes were modeled separately as five-level ordinal variables considering PA guidelines. Using ordinal logistic regression models adjusting for age, sex, health status, smoking, and body mass index, we found that living within one kilometer of at least one PA destination was associated with significantly increased odds of engaging in higher levels of all four PA components (odds ratios: 1.17-1.20, all Ps < 0.01). After adjusting for social factors (race, education, income), associations were attenuated and no longer significant. When evaluating barriers to PA, social determinants of health should be considered, in addition to proximity of PA destinations.

